# Toll-like Receptor 3 Regulates Neural Stem Cell Proliferation by Modulating the Sonic Hedgehog Pathway

**DOI:** 10.1371/journal.pone.0026766

**Published:** 2011-10-25

**Authors:** Kavitha Yaddanapudi, Joari De Miranda, Mady Hornig, W. Ian Lipkin

**Affiliations:** Center for Infection and Immunity, Mailman School of Public Health, Columbia University, New York, New York, United States of America; The University of Hong Kong, Hong Kong

## Abstract

Toll-like receptor 3 (TLR3) signaling has been implicated in neural stem/precursor cell (NPC) proliferation. However, the molecular mechanisms involved, and their relationship to classical TLR-mediated innate immune pathways, remain unknown. Here, we report investigation of the mechanics of TLR3 signaling in neurospheres comprised of epidermal growth factor (EGF)-responsive NPC isolated from murine embryonic cerebral cortex of C57BL/6 (WT) or TLR3 deficient (TLR3^−/−^) mice. Our data indicate that the TLR3 ligand polyinosinic-polycytidylic acid (PIC) negatively regulates NPC proliferation by inhibiting Sonic Hedgehog (Shh) signaling, that PIC induces apoptosis in association with inhibition of Ras-ERK signaling and elevated expression of Fas, and that these effects are TLR3-dependent, suggesting convergent signaling between the Shh and TLR3 pathways.

## Introduction

PIC is a synthetic analog of viral double-stranded (ds) RNA that activates immune responses through two dsRNA sensors, TLR3 and melanoma differentiation-associated protein 5 (MDA5). TLR3 senses PIC that has been internalized by endocytosis [1], [2]. Upon binding PIC, TLR3 signals through the adaptor protein, Toll/IL-1 resistance domain-containing adaptor-inducing IFN-β (TRIF). Interaction with the adaptor activates an array of transcription factors, including IFN regulatory factor (IRF) 3, IRF7, IRF1, and NFκB. These factors induce the expression of genes encoding type I interferon (IFN, i.e., IFN-α and IFN-β) and proinflammatory cytokines [3], [4].

Recent evidence suggests that TLR3 plays a role in neural development. TLR3 protein is present in mammalian brain cells in early embryonic stages of development and serves as a negative modulator of early embryonic NPC proliferation [5] and axonal growth [6]. In a mouse model of prenatal virus infection, we recently demonstrated that PIC-induced TLR3 activation inhibits embryonic NPC proliferation and decreases the number of neurons populating the upper layers of the cortex [7].

Single-cell suspensions of neural stem cells can be isolated from the embryonic telencephalon and propagated *in vitro* as suspended, spherical aggregates called neurospheres [8], [9]. Epidermal growth factor (EGF)-responsive NPC in neurospheres express the neuroepithelial stem cell marker, nestin, and are derived from rapidly-cycling, radial glia (RG) in the embryonic telencephalon. Neural stem cells can give rise to all three major cell types of the central nervous system: neurons, oligodendrocytes, and astrocytes [10], [11]. Accordingly, neurospheres contain a mixture of multipotent stem cells, proliferating precursor cells, postmitotic neurons and glia [12], [13], [14]. Primary neurospheres can be clonally passaged *in vitro* and provide a useful tool for analysis of the proliferation and self-renewal capacity of neural stem and precursor cells.

Regulation of the number of stem and precursor cells generated during neural development is important for control of brain size [15], [16]. Shh signaling has been implicated in cell proliferation and growth of embryonic and postnatal dorsal brain [17], [18], [19], [20], [21], [22], [23]. Shh is a member of the hedgehog family of secreted glycoproteins that binds the cell surface receptor Patched (Ptch). Binding of Shh and related ligands to Ptch abrogates its inhibition of the G-protein-coupled receptor, Smoothened (Smo), resulting in increased expression of Gli1 zinc-finger transcription factors [24], [25]. Three Gli proteins participate in the mediation of Shh signaling: Gli1 and Gli2 function as transcription activators, whereas the truncated form of Gli3, Gli3R, acts as a repressor [26], [27], [28], [29]. Shh-Gli signaling induces formation of Gli activators (Gli1, Gli2) that are imported into the nucleus to transactivate target genes. Multiple effects of Shh signaling on cyclin-dependent kinases (Cdks), Cdk inhibitors, cyclins, N-myc or the transcription factor, E2F, acting at different points of the cell cycle, may account for the proliferative effects of Shh [30], [31], [32], [33], [34].

In mammals, Shh is the only hedgehog family member expressed in the normal central nervous system [35]. Shh expression is layer-specific in perinatal neocortex and tectum. In the embryonic telencephalon, Shh is expressed within the mantle of the medial ganglionic eminence, the preoptic area and the amygdala [35], [36], [37]. Shh secreted from differentiated cells in the cortex can affect Gli1-positive, cycling precursor cells located at a distance; in addition, Shh can also be produced by the precursors themselves [18], [38], [39]. Genetic loss-of-function and knock-in studies wherein Gli genes have been ablated and then reintroduced have shown that the Shh-Gli pathway controls the growth and dorsal-ventral patterning of brain structures by regulating proliferation of neural stem cells through EGF signaling [40], [41], [42], [43], [44].

Shh signaling also plays a role in inducing apoptosis. Signaling via Ptch, a 12–transmembrane domain receptor of Shh, induces caspase-mediated apoptosis in neuroepithelial cells. The intracellular domain of Ptch harbors a cleavage site for caspase 3; cleavage at this site by caspase 3 exposes the proapoptotic domain of the receptor. *In vitro* treatment of neuroepithelial cells with recombinant Shh blocks Ptch-induced cell death [45], [46].

Here we demonstrate that: (i) PIC negatively regulates NPC proliferation by inhibiting Shh signaling, (ii) PIC induces apoptosis in association with inhibition of Ras-ERK signaling and elevated expression of Fas, and (iii) that these effects depend on TLR3 activation.

## Results

### Phenotypic analysis of EGF-responsive primary cortical neurospheres

**Figure 1:**
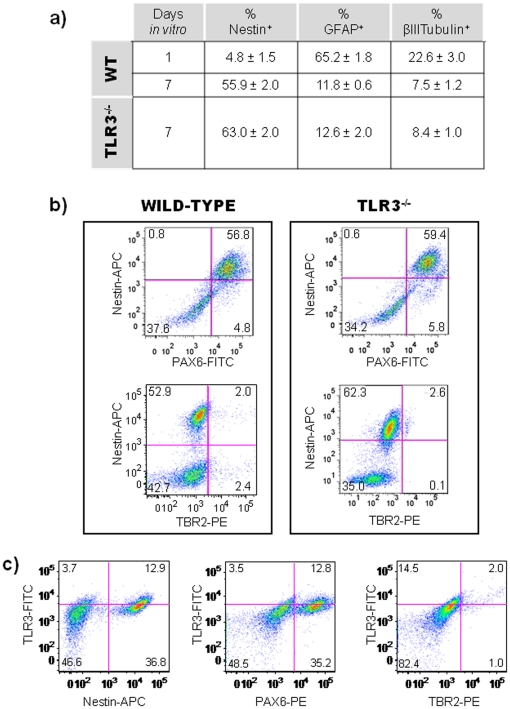
Phenotypic analysis of EGF-responsive primary cortical neurospheres. Primary neurospheres were obtained from cerebral cortex cells isolated from WT and TLR3^−/−^ embryos at GD14. Cell populations in neurospheres were assayed by using flow cytometry. (**a**) Percentages of nestin-, GFAP- and Tubulin-β-III-expressing cells in neurospheres cultured for 24 hours or 7 days in proliferation medium supplemented with 20 ng/ml of EGF. Three independent experiments were performed with neurosphere-derived cells isolated from WT and TLR3^−/−^ embryos (n = 12). Results are expressed as percentages of total gated cells. Values represent mean ± SEM. (**b**) Dot plots of PAX6, TBR2, and nestin expression in cells derived from WT and TLR3^−/−^ cortical neurospheres cultured for 7 days. Numbers in quadrants represent the percentages of each subpopulation. Three independent cell culture assays were performed with cells isolated from WT and TLR3^−/−^ embryos (n = 12); data from one representative assay is shown. (**c**) Dot plots of TLR3 expression in nestin^+^, PAX6^+^ and TBR2^+^ WT cortical neurosphere cells cultured for 7 days. Numbers in quadrants represent the percentages of each subpopulation. Three independent cell culture assays were performed with cells isolated from WT embryos (n = 12); data from one representative assay is shown.

Neurospheres were generated from dissociated cerebral cortex obtained from Wild-type (WT) or TLR3 knockout (TLR3^−/−^) C57BL/6 mouse embryos at GD14. The cellular composition of neurospheres was surveyed by FACS after culture for 1 or 7 days in proliferation medium supplemented with EGF (20 ng/ml). After 1 day of culture, 65.2 ± 1.8% of WT cells expressed GFAP, a marker for astrocytes; 22.6 ± 3.0% expressed tubulin-β-III, a marker for young postmitotic neurons; 4.8 ± 1.5% expressed nestin, a marker for neuroepithelial stem cells (**Fig. 1a**; values represent mean ± SEM) [47], [48], [49]. After 7 days, the percentage of GFAP^+^ and tubulin-β-III^+^ cells in WT neurospheres decreased to 11.8 ± 0.6% and 7.5% ± 1.2%, respectively, whereas the percentage of nestin^+^ precursors increased to 55.9 ± 2.0% (**Fig. 1a**; values represent mean ± SEM**).** We observed similar percentages of cells in day 7 neurosphere cultures derived from TLR3 knockout mice (TLR3^−/−^). After 7 days of culture, 63.0±2.0% of TLR3^−/−^ neurosphere cells expressed nestin; 12.6±2.0% expressed GFAP; and 8.4±1.0% expressed tubulin-β-III, suggesting that neurospheres derived from TLR3^−/−^ mice are equally responsive to EGF (**Fig. 1a**; values represent mean ± SEM).

To further characterize the phenotype of nestin^+^ NPC, day 7 WT and TLR3^−/−^ neurosphere cells were analyzed by FACS for expression of PAX6 and TBR2, two transcription factors expressed *in vivo* by NPC during mid- and late cortical neurogenesis [50], [51], [52]. 46.2 ± 2.8% of WT and 62.3 ± 2.0% of TLR3^−/−^ precursor cells co-expressed nestin and PAX6; 2.2 ± 0.07% WT and 2.6 ± 0.08% of TLR3^−/−^ cells co-expressed nestin and TBR2. PAX6 is a radial glial (RG) marker; thus, these results indicate that the majority of the nestin^+^ neurosphere-derived cells in these dorsal telencephalon cultures are self-renewing NPC (**Fig. 1b**; values represent mean ± SEM**)**. In WT neurosphere cells, intracellular expression of TLR3 was observed (in nestin^+^, PAX6^+^, and TBR2^+^ NPC populations (**Fig. 1c**).

The neurosphere culture model contains cells at various stages of differentiation [8], [9]. Whereas nestin-positive precursor cells are typically located toward the outside of the sphere, GFAP-positive cells are found in the center. Tubulin-β-III-positive neurons are less common and tend to be evenly distributed [53]. In our experiments, neurospheres were detected after one week of culture of WT and TLR3^−/−^ GD14 cortices (**Fig. S1**). The number of nestin-positive cells was decreased by 50% in secondary versus primary WT neurospheres (**Fig. S2**) and decreased continuously with subsequent passages (data not shown) [54]. Thus, our experiments were performed with primary neurospheres wherein nestin expression was maximal. When dissociated and transferred to differentiating conditions for 7 days, WT primary neurospheres lost their spherical shape and flattened to form monolayers (**Fig. S3a**). FACS analysis using cell lineage-specific antisera showed that WT neurospheres were multipotent and capable of giving rise to neurons, astrocytes, and oligodendrocytes. During differentiation, WT cells expressed GFAP, tubulin-β-III, and O4, markers characteristic for astrocytes, neurons and oligodendrocyte populations, respectively (**Fig. S3b–f**).

### PIC negatively regulates neurosphere-derived NPC proliferation in a TLR3-dependent manner

**Figure 2:**
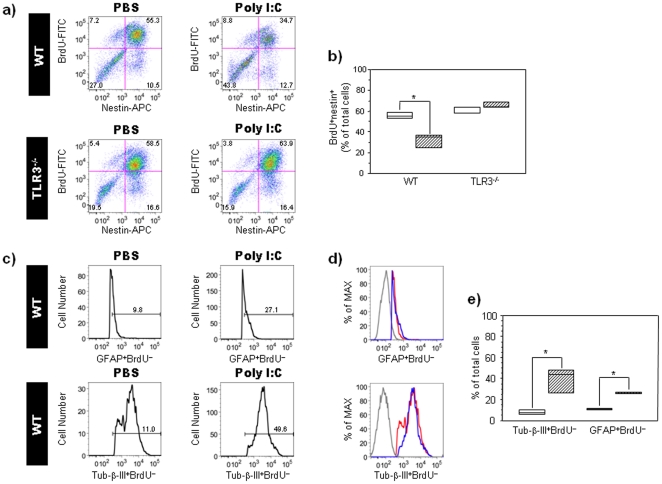
PIC negatively regulates neurosphere-derived NPC proliferation in a TLR3-dependent manner. Proliferative response of primary cortical neurospheres obtained from WT and TLR3^−/−^ embryos at GD14. Neurospheres were maintained in EGF-containing (20 ng/ml) medium for 7 days and treated with either PBS or PIC for 24 hours. BrdU was added for the last 12 hours of the culture. (**a**) Dot plots showing BrdU-labeled nestin^+^ cells in PBS- and PIC-treated neurospheres derived from WT and TLR3^−/−^ mice. Numbers in quadrants represent the percentages of each subpopulation. Three independent cell culture assays were performed with cells isolated from embryos (n = 12); data from one representative assay is shown. (**b**) Box plots showing percentages of BrdU^+^nestin^+^ cells in PBS- and PIC-treated neurospheres derived from WT and TLR3^−/−^embryos. Results are expressed as percentages of total cells (* p<0.05; relative to PBS control group; Mann-Whitney U test). (**c**) Histograms showing BrdU-negative GFAP^+^ and Tubulin-β-III^+^ cells in PBS- and PIC-treated neurospheres derived from WT mice. Numbers in the graph represent the percentages of each subpopulation. (**d**) Histograms showing the mean fluorescence intensity of GFAP and Tubulin-β-III expression in BrdU-negative cells from PBS- and PIC-treated neurospheres obtained from WT mice. The mean fluorescence intensity is represented as a percentage of the maximum expression: red line: PBS-treated cells; blue line: PIC-treated cells; and gray line: isotype control. (e) Box plots of the percentages of Tubulin-β-III^+^BrdU^−^ and GFAP^+^BrdU^−^ cells from PBS- and PIC-treated neurospheres derived from WT embryos. Results are expressed as percentages of total cells (*, p<0.05; relative to PBS control group; Mann-Whitney U test). Height of box plots show interquartile range; horizontal line, median. Due to the low numbers involved in the flow cytometry experiments, the box plot upper and lower interquartile ranges also represent the maximum and minimum scores. White bars: PBS-treated mice, hatched bars: PIC-treated mice.

To assess the direct role of TLR3 signaling on NPC proliferation, we used primary cortical neurosphere cultures obtained from GD14 WT and TLR3-deficient (TLR3^−/−^) embryos. Neurospheres maintained in proliferation media for 7 days were treated with the TLR3 ligand, PIC (50 µg/ml), for 24 hours; BrdU was added for the final 12 hours of the culture. Proliferative responses of neurosphere-derived cells were assessed by flow cytometric measurement of labeled BrdU. Our results indicate that BrdU was incorporated into the majority of nestin^+^ cells, and that PIC exposure was associated with a reduced percentage of BrdU^+^nestin^+^ precursor cells in WT neurosphere cultures as compared to PBS-treated WT control cultures (n = 12; Mann-Whitney U, *p* = 0.04; relative to PBS-treated WT control group; **Fig. 2a, b**). PIC treatment resulted in a concomitant increase in the percentage of BrdU-negative GFAP^+^ and Tubulin-β-III^+^ subpopulations in the WT neurospheres (n = 12; Mann-Whitney U, *p* = 0.04; relative to PBS-treated WT control group; **Fig. 2c, e**). No significant differences were observed in expression levels of GFAP and Tubulin-β-III markers in WT neurospheres (n = 12; Mann-Whitney U, *p* = 0.29; **Fig. 2d**). Neurosphere cultures derived from TLR3^−/−^ mice were used to assess the role of TLR3 signaling in the PIC-mediated inhibition of NPC proliferation. No differences were observed in the percentage of BrdU^+^nestin^+^ precursor cells in PIC-treated TLR3^−/−^ neurospheres as compared to PBS-treated TLR3^−/−^ control cultures (n = 12; Mann-Whitney U, *p* = 0.26; **Fig. 2a, b**). Taken together, these results implicate TLR3 signaling in PIC-induced impairment of proliferation of EGF-responsive NPC in cortical neurospheres.

### PIC decreases neurosphere-derived neural precursor cell proliferation by inhibiting Shh signaling

**Figure 3:**
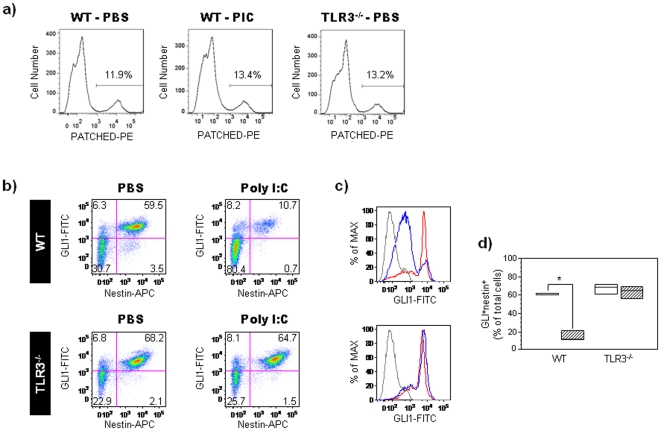
PIC decreases neurosphere-derived NPC proliferation by inhibiting Shh signaling. Primary neurospheres were obtained from cerebral cortex cells isolated from WT and TLR3^−/−^ embryos at GD14. Neurospheres were cultured for 7 days in the presence of EGF (20 ng/ml) and treated with either PBS or PIC for the last 24 hours of the culture period. (**a**) Representative histograms showing Patched expression in nestin-positive cells from PBS- and PIC-treated neurospheres obtained from WT and TLR3^−/−^ mice. Numbers in the graphs represent the percentage of nestin^+^ cells expressing Patched. Three independent cell culture assays were performed with cells isolated from embryos (n = 12); data from one representative assay is shown. (**b**) Dot plots showing Gli1^+^nestin^+^ cells in PBS- and PIC-treated neurospheres derived from WT and TLR3^−/−^ mice. Numbers in quadrants represent the percentages of each subpopulation. Three independent cell culture assays were performed with cells isolated from embryos (n = 12); data from one representative assay is shown. (**c**) Histograms showing the mean fluorescence intensity of Gli1 expression in PBS- and PIC-treated neurospheres obtained from WT and TLR3^−/−^ embryos. The mean fluorescence intensity is represented as a percentage of the maximum expression: red line: PBS-treated cells, blue line: PIC-treated cells, and gray line: isotype control. (**d**) Box plot showing percentages of Gli1^+^nestin^+^ cells in PBS- and PIC-treated neurospheres derived from WT and TLR3^−/−^ embryos. Results are expressed as percentages of total cells (*, p<0.05 relative to PBS control group; Mann-Whitney U test). Height of box plot shows interquartile range; horizontal line, median. Due to the low numbers involved in the flow cytometry experiments, the box plot upper and lower interquartile ranges also represent the maximum and minimum scores. White bars: PBS-treated mice, hatched bars: PIC-treated mice.

Shh signaling regulates the proliferation of EGF-responsive precursor cells in the developing mouse neocortex [41]. To test whether Shh signaling is active in the EGF-responsive precursor cells found in neurospheres, we examined the expression of Shh pathway receptors and targets in 7-day cultures from GD14 embryos. FACS analysis revealed that WT and TLR3^−/−^ nestin^+^ neurosphere cells expressed the membrane-associated Shh receptor, Ptch (**Fig. 3a**). No change in the percentage of nestin^+^ Ptch^+^ cells was observed with PIC treatment. 11.0–15.0% of nestin^+^ cells expressed Ptch in both PBS- and PIC-treated WT cells (**Fig. 3a**). WT and TLR3^−/−^ Nestin^+^ cells expressed the Shh-regulated transcription factor, Gli1 (**Fig. 3b, c**). To test the effect of PIC on Shh signaling in EGF-responsive NPC, GD14 WT and TLR3^−/−^ cortical neurospheres were grown in EGF-supplemented medium for 7 days, and treated with either PIC or PBS for the last 24 hours of the culture period. FACS analysis of WT neurospheres indicated that PIC treatment decreased the percentage of Gli1^+^nestin^+^ cells (n = 12; Mann-Whitney U, *p* = 0.04; relative to PBS-treated WT control group; **Fig. 3b, d**) and the mean fluorescent intensity of Gli1 expression (n = 12; Mann-Whitney U, *p* = 0.04; relative to PBS-treated WT control group; **Fig. 3c**). In contrast, no differences were observed between TLR3^−/−^ cells treated with PIC or PBS (n = 12; Mann-Whitney U, *p* = 0.51; relative to PBS-treated TLR3^−/−^ control group; **Fig. 3b–d**). Together, these results suggest that PIC-mediated inhibition of Shh signaling in nestin^+^ precursor cells may be occurring downstream to Ptch and that this inhibition requires TLR3 expression.

### Shh pathway activation reverses PIC-mediated inhibition of neurosphere-derived NPC proliferation

**Figure 4:**
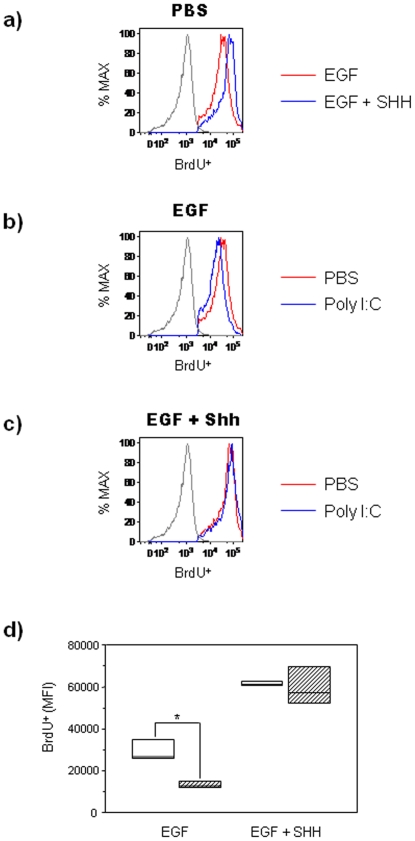
Shh pathway activation reverses PIC-mediated inhibition of neurosphere-derived NPC proliferation. Primary neurospheres were obtained from cerebral cortex cells isolated from WT embryos at GD14. Neurospheres were cultured for 7 days in the presence of EGF (5 ng/ml) and treated with PBS or PIC for 24 hours. BrdU was added for the last 12 hours of the culture. (**a**) Proliferative response of neurospheres in EGF-containing medium with or without added recombinant Shh protein (5 nM). Representative histograms showing the mean fluorescence intensity of BrdU-labeled cells represented as a percentage of maximum BrdU incorporation in WT neurospheres: green line: EGF, red line: EGF + Shh, and gray line: isotype control. (**b**) Representative histograms showing the mean fluorescence intensity of BrdU labeling in PBS- or PIC-treated neurospheres cultured in EGF. (**c**) Representative histograms showing the mean fluorescence intensity of BrdU labeling in PBS- or PIC-treated neurospheres cultured in the presence of EGF (5 ng/ml) and Shh (5 nM): red line: PBS-treated cells, blue line: PIC-treated cells, and gray line: isotype control. (**d**) Box plot showing the mean fluorescent intensity of BrdU-labeled cells in PBS- and PIC-treated WT neurospheres cultured in the presence of EGF and EGF + Shh. Results are expressed as percentages of maximum expression (*, p<0.05 relative to PBS control group; Mann-Whitney U test). Three independent cell culture assays were performed with cells isolated from embryos (n = 12). Height of box plot shows inter-quartile range; horizontal line, median. Due to the low numbers involved in the flow cytometry experiments, the box plot upper and lower interquartile ranges also represent the maximum and minimum scores. White bars: PBS-treated mice, hatched bars: PIC-treated mice.

**Figure 5:**
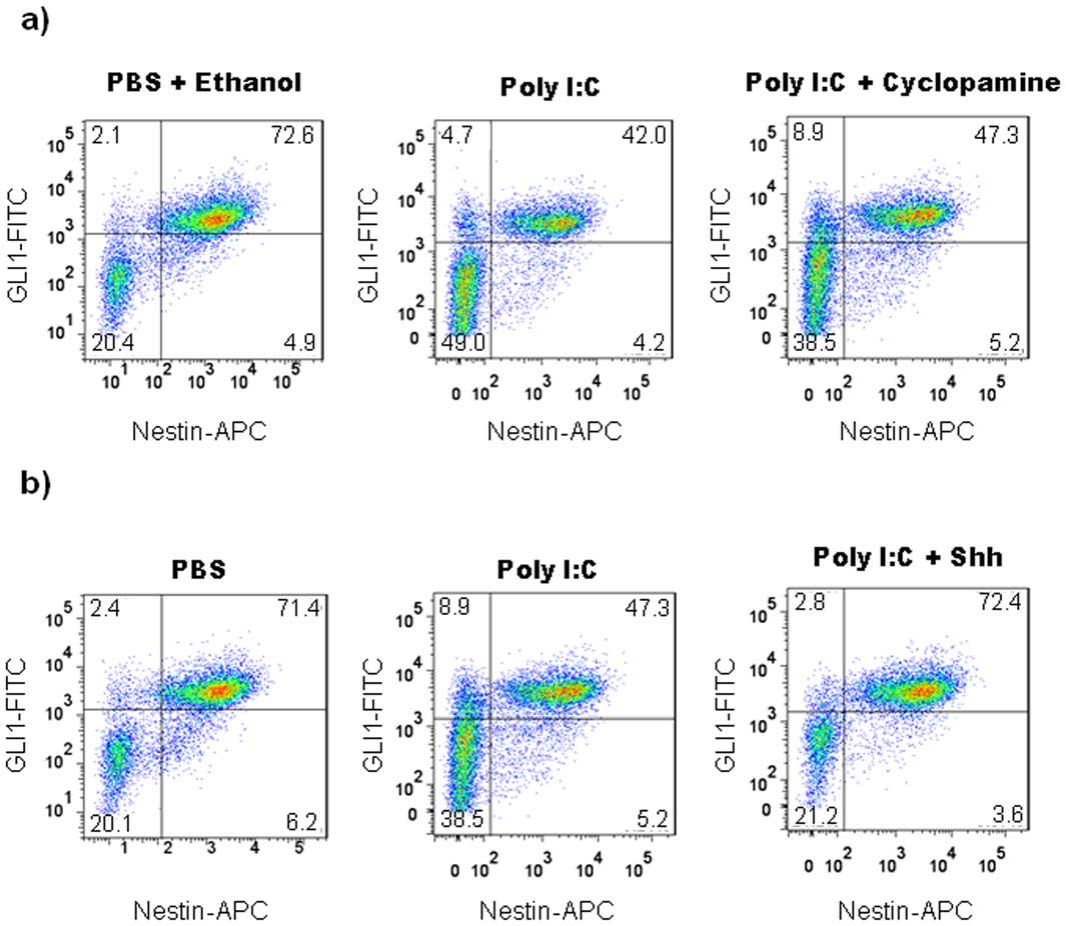
Shh pathway activation reverses PIC-mediated inhibition of GLI1 expression in WT NPCs. Primary neurospheres were obtained from cerebral cortex cells isolated from WT embryos at GD14. Neurospheres were cultured for 7 days with EGF (5 ng/ml) either in the presence or in the absence of cyclopamine (5 µM) and treated with PIC for the last 24 hours. The control group includes cells cultured with EGF (5 ng/ml) and ethanol and treated with PBS for the last 24 hours (vehicle control group) (**a**) Dot plots showing Gli1^+^nestin^+^ cells in PIC-treated WT neurospheres cultured either in the presence or absence of cyclopamine and vehicle control group. Numbers in quadrants represent the percentages of each subpopulation. Three independent cell culture assays were performed with cells isolated from embryos (n = 12); data from one representative assay is shown. (**b**) Dot plots showing Gli1^+^nestin^+^ cells expression in PBS- and PIC-treated neurospheres incubated in EGF-containing medium (5 ng/ml) with or without added recombinant Shh protein (5 nM). Numbers in quadrants represent the percentages of each subpopulation. Three independent cell culture assays were performed with cells isolated from embryos (n = 12); data from one representative assay is shown.

Induction of Shh signaling with recombinant Shh protein regulates proliferation of neural stem cells [41]. GD14 WT-derived primary cortical neurospheres grown for 7 days in media containing 5 ng/ml of EGF supplemented with 5 nM recombinant Shh showed increased proliferation as measured by BrdU labeling (**Fig. 4a**). PIC treatment inhibited BrdU labeling in WT neurospheres cultured in 5 ng/ml of EGF (n = 12; Mann-Whitney U, *p* = 0.04; relative to PBS-treated WT control group; **Fig. 4b, d**). Recombinant Shh treatment in the presence of 5 ng/ml of EGF abrogated the PIC-mediated reduction in BrdU labeling (n = 12; Mann-Whitney U, *p* = 0.52; relative to Shh and PBS-treated WT control group; **Fig. 4c, d** **)**. Under culture conditions with a higher concentration of EGF (20 ng/ml) in the media, Shh did not have this effect (data not shown), presumably because stimuli for proliferation were already maximal [41]. These results indicate that PIC-mediated inhibition of NPC proliferation can be abrogated by stimulating the Shh pathway under culture conditions wherein EGF concentration is sub maximal. To test for direct evidence that PIC effect is due to Shh signaling inhibition, neurosphere cultures were treated with cyclopamine, a small molecule inhibitor of Shh signaling. Cyclopamine is a steroidal alkaloid that specifically inhibits Shh pathway activation by binding directly to the multipass transmembrane protein, Smo, and regulating its function [55]. WT cortical neurospheres were grown in 5 ng/ml of EGF supplemented with 5 µM of cyclopamine or ethanol (vehicle control group) for 7 days, and treated with PIC or PBS for the last 24 hours of the culture period. FACS analysis of WT neurospheres indicated that PIC treatment in the presence of cyclopamine and EGF decreased the percentage of Gli1^+^nestin^+^ cells when compared to PBS/ethanol-treated vehicle control group (**Fig. 5a**). PIC treatment in the presence of cyclopamine and EGF did not result in any additive effects in the inhibition of Gli1 in comparison to cells that were treated with PIC alone (**Fig. 5a**). These observations suggest: (i) a lack of synergy between PIC and cyclopamine on Shh signaling, and (ii) in WT neurosphere cells, PIC-mediated effects in the Shh signaling pathway may be on effectors that are downstream to Ptch and Smo. Furthermore, no differences in percentages of Gli1^+^nestin^+^ cells were observed in PIC-treated WT cells cultured in the presence of recombinant Shh (5 nM) and EGF (5 ng/ml) in comparison to cells from the PBS-treated WT control group (**Fig. 5b**), confirming our earlier observation that in neurosphere cultures, under conditions of sub-maximal EGF stimulation, recombinant Shh treatment abrogates the PIC-mediated effects on Shh signaling.

### PIC induces apoptosis in primary cortical neurospheres

**Figure 6:**
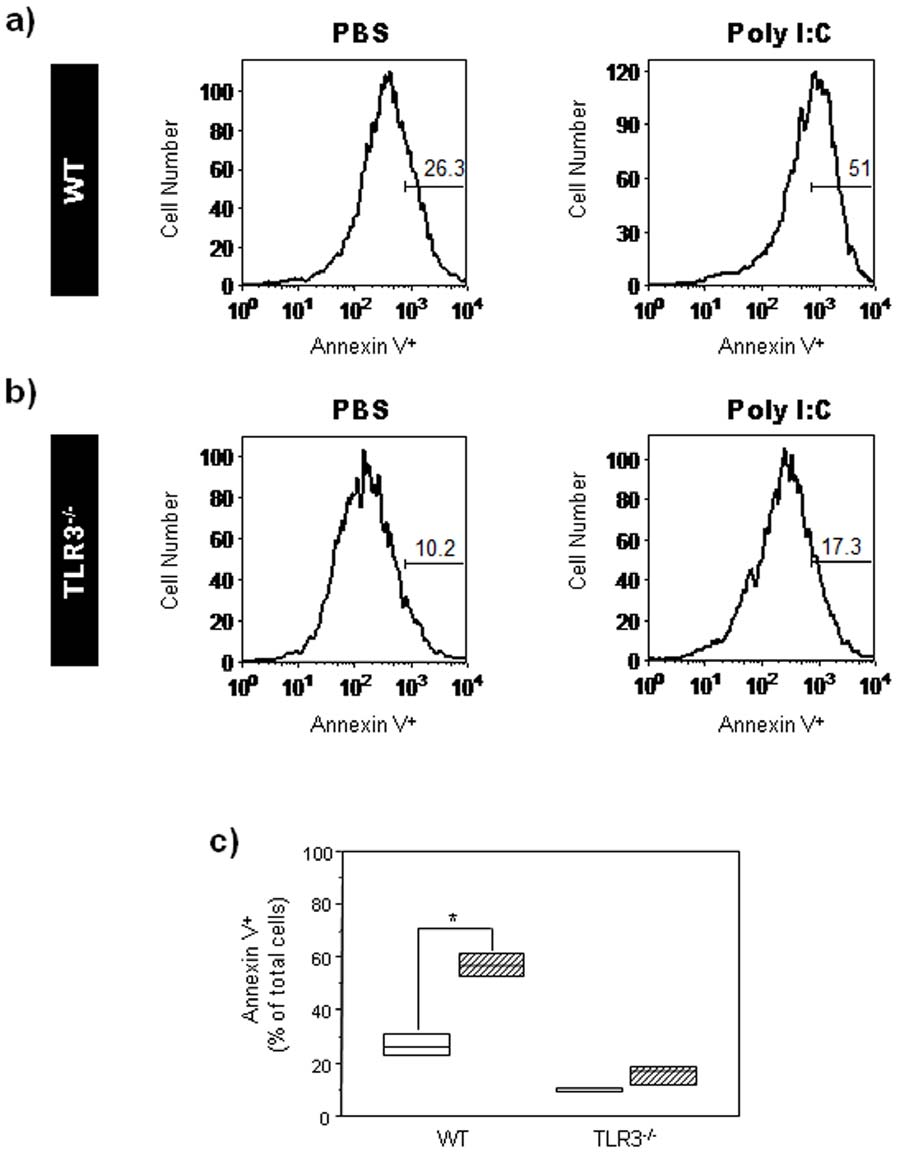
PIC induces apoptosis in primary cortical neurospheres. Primary neurospheres were obtained from cerebral cortex cells isolated from WT and TLR3^−/−^ embryos at GD14. Neurospheres were cultured for 7 days in the presence of EGF (20 ng/ml) and treated with PBS or PIC for 24 hours. (**a, b**) Representative histograms showing Annexin V-positive cells in PBS- or PIC-treated WT or TLR3^−/−^ neurospheres. Numbers in gates represent the percentages of Annexin V^+^ cells. Three independent cell culture assays were performed with cells isolated from embryos (n = 12); data from one representative assay is shown. (**c**) Box plot showing the percentages of Annexin V^+^ cells in PBS- and PIC-treated neurospheres derived from WT and TLR3^−/−^ embryos. Results are expressed as percentages of total cells (*, p<0.05 relative to PBS control group; Mann-Whitney U test). Height of box plot shows inter-quartile range; horizontal line, median. Due to the low numbers involved in the flow cytometry experiments, the box plot upper and lower interquartile ranges also represent the maximum and minimum scores. White bars: PBS-treated mice, hatched bars: PIC-treated mice.

To test whether PIC altered viability of NPC, we measured apoptosis in GD14 WT and TLR3^−/−^ neurospheres after 7 days of culture in proliferation medium supplemented with EGF (20 ng/ml) that included 24 hours of treatment with PIC or PBS. PIC resulted in a close to two-fold increase in the percentage of annexin V^+^ cells in WT neurospheres (n = 12; Mann-Whitney U, *p* = 0.04; relative to PBS-treated WT control group; **Fig. 6a, c**). In contrast, no significant differences were observed between PIC- and PBS-treated TLR3^−/−^ neurospheres (n = 12; Mann-Whitney U, *p* = 0.27; relative to PBS-treated TLR3^−/−^ control group; **Fig. 6b, c**). These results indicate that PIC augments apoptosis in NPC and that the effect is TLR3-dependent.

### PIC prevents EGF/Shh-mediated ERK activation and induces Fas expression in primary cortical neurospheres

**Figure 7:**
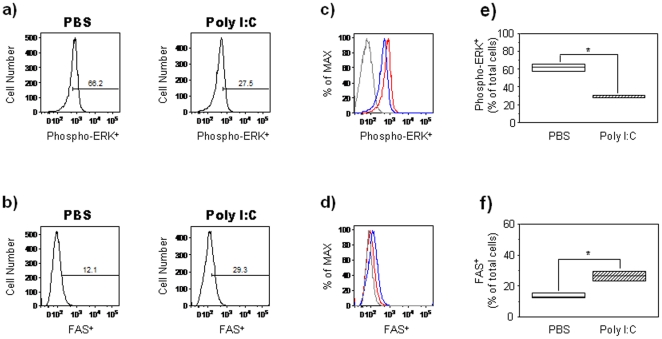
PIC prevents EGF/Shh–mediated ERK activation and induces Fas expression in primary cortical neurospheres. Primary neurospheres were obtained from cerebral cortex cells isolated from WT embryos at GD14. Neurospheres were cultured for 7 days in the presence of EGF (20 ng/ml) and treated with PBS or PIC for 24 hours. (**a, b**) Representative histograms showing phospho-ERK and Fas-positive cells in PBS- or PIC-treated WT neurospheres. Numbers in gates represent the percentages of phospho-ERK^+^ and Fas^+^ cells. Three independent cell culture assays were performed with cells isolated from embryos (n = 12); data from one representative assay is shown. (**c, d**) Representative histogram showing the mean fluorescence intensity of phospho-ERK and Fas expression in PBS- and PIC-treated WT neurospheres. The mean fluorescence intensity is represented as a percentage of the maximum expression, ——, PBS-treated cells; ——, PIC-treated cells; and ——, isotype control. (**e, f**) Box plots showing the percentages of phospho-ERK^+^ and Fas^+^ cells in PBS- and PIC-treated neurospheres. Results are expressed as percentages of total cells (*, p<0.05 relative to PBS control group; Mann-Whitney U test). Height of box plots show interquartile range; horizontal line, median. Due to the low numbers involved in the flow cytometry experiments, the box plot upper and lower interquartile ranges also represent the maximum and minimum scores. White bars: PBS-treated mice, hatched bars: PIC-treated mice.

**Figure 8:**
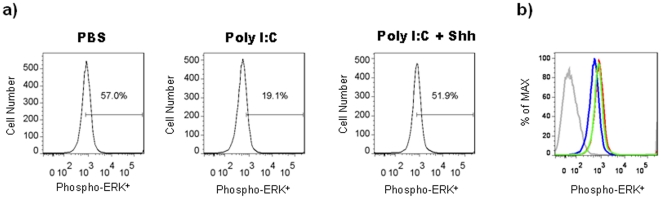
Shh pathway activation reverses PIC-induced ERK activation. Primary neurospheres were obtained from cerebral cortex cells isolated from WT embryos at GD14. Neurospheres were cultured for 7 days in the presence of EGF (5 ng/ml) and in the presence or absence of recombinant Shh protein (5 nM), and treated with PBS or PIC for the last 24 hours of the culture. (**a**) Representative histograms showing percentage of cells expressing phospho-ERK in WT neurospheres. Numbers in gates represent the percentages of phospho-ERK^+^ cells. Three independent cell culture assays were performed with cells isolated from embryos (n = 12); data from one representative assay is shown. (**b**) Histogram showing the mean fluorescence intensity of phospho-ERK expression in PBS- and PIC-treated WT neurospheres cultured in the presence or absence of recombinant Shh. The mean fluorescence intensity is represented as a percentage of the maximum expression: red line: EGF and PBS-treated cells, blue line: EGF and PIC-treated cells, green line: EGF + Shh and PIC-treated cells supplemented with Shh, and gray line: isotype control.

To elucidate the molecular basis by which PIC mediates inhibition of Shh pathway activation, we examined the effects of PIC on the Ras-ERK pathway, a downstream effector of Shh and EGF signaling in GD14 WT primary cortical neurospheres cultured in the presence of EGF. PIC treatment decreased the percentage of neurosphere-derived cells expressing phosphorylated-ERK, the active form of the protein (n = 12; Mann-Whitney U, *p* = 0.02; relative to PBS-treated WT control group; **Fig. 7a, e**). The mean fluorescent intensity of phosphorylated-ERK was also reduced in PIC-treated cells (n = 12; Mann-Whitney U, *p* = 0.04; relative to PBS-treated WT control group; **Fig. 7c**). Consistent with studies reporting regulation of Fas (CD95) expression via the Ras-ERK pathway [56], [57], [58] Fas expression was elevated in WT cortical NPC treated with PIC (n = 12; Mann-Whitney U, *p* = 0.02; relative to PBS-treated WT control group; **Fig. 7b, d, f**). Collectively, these results suggest that inhibition of Ras-ERK signaling by PIC may lead to elevated levels of Fas and Fas-mediated apoptosis in NPC. To elucidate that Poly IC-mediated effects are dependent on Shh, neurosphere cultures were grown for 7 days in media supplemented with recombinant Shh (5 nM) in the presence of sub-maximal concentration of EGF (5 ng/ml) and treated with PIC or PBS as described above. Recombinant Shh pre-treatment in the presence of EGF abrogated PIC-mediated inhibition of phospho-ERK expression in WT neurosphere cells as compared with PIC-treated cells cultured in the presence of EGF alone (**Fig. 8a, b**).

## Discussion

The majority of EGF-responsive nestin^+^ cells in the cortical neurosphere model correspond to the rapidly-cycling, PAX6^+^ RG stem cells and support the hypothesis that stem cells are contained within the neuroepithelial-radial glia-astrocyte lineage [59]. We recently reported that PAX6^+^ RG cells express TLR3 in embryonic neocortex at GD18 [7]. Results reported here confirm that TLR3 receptors are expressed on NPC and regulate PIC signaling. PIC treatment inhibited the proliferation of NPC and increased the ratio of GFAP^+^- and βIII-tubulin^+^-differentiated subpopulations in primary WT neurospheres. Although we observed comparable NPC multipotency in control and PIC-treated WT neurospheres (data not shown), we cannot ascertain from current data whether PIC induces NPC differentiation.

**Figure 9:**
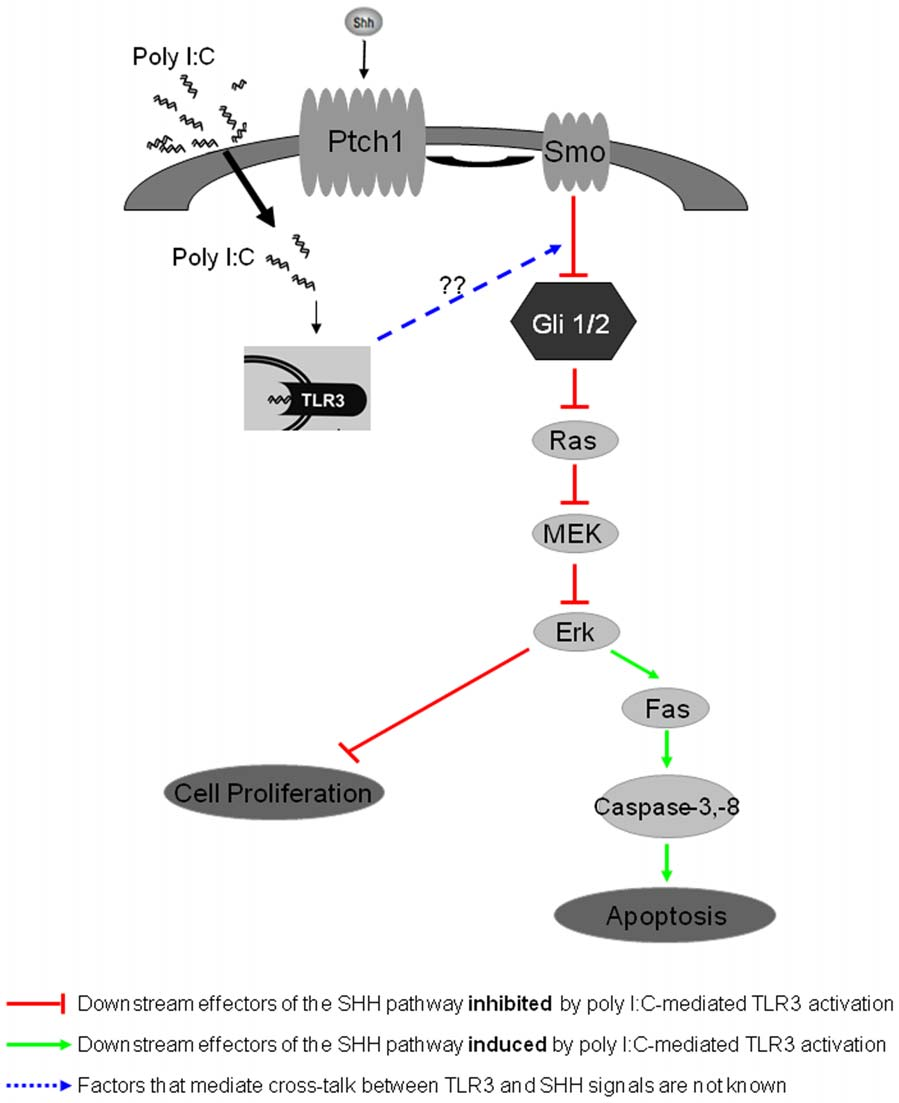
PIC treatment inhibits the EGF-Shh-mediated Ras-ERK signaling pathway in primary cortical neurospheres. Shh binds to the cell surface receptor Ptch, relieves the inhibition of Smo and triggers Shh signaling. Activation of the Shh pathway results in increased levels of Gli1 expression and activation of the downstream Ras-MEK-ERK pathway, causing cell proliferation. In WT neurospheres cultured in the presence of EGF, PIC signals via TLR3 to inhibit Gli1 expression and interfere with MEK and ERK phosphorylation. Inhibition of MEK-ERK activation results in the decreased proliferation and induced Fas expression and apoptosis observed after PIC treatment of primary cortical neurospheres.

The Ras-MEK-ERK signaling pathway is involved in cell proliferation, apoptosis and differentiation. EGF stimulates cell proliferation via the Ras-MEK-ERK signaling cascade [60], [61], [62]. Furthermore, Ras-ERK activation is implicated both in the maintenance of tumor stem cells [63], and in regulation of Fas transcription [56], [64]. Our results in EGF-responsive NPC indicate that Shh signaling machinery is present in nestin^+^ NPC in primary cortical neurospheres. PIC treatment inhibited Shh signaling in WT primary neurospheres, resulting in reduced proliferation of NPC. PIC also induced apoptosis in the WT neurospheres. In Figure 9, we present a model of PIC-mediated effects on NPC. PIC inhibits the Shh-mediated Ras-MEK-ERK signaling pathway, resulting in reduced MEK (data not shown) and ERK phosphorylation and increased Fas expression. Thus, PIC-mediated inhibition of Ras-MEK-ERK activation may contribute to both suppression of proliferation and induction of apoptosis.

PIC signals via a TLR3-dependent NFκB pathway to induce IFN-α, IFN-β and proinflammatory cytokines [3], [65]. Li and colleagues have shown that IFN-α inhibits the Shh-activated Ras-MEK-ERK pathway and induces apoptosis in human cancer cells [46]. Our experiments with neurospheres from TLR3-deficient mice indicate that PIC interferes with Shh signaling and NPC proliferation in a TLR3-dependent manner: PIC treatment of TLR3^−/−^ neurospheres neither induced apoptosis nor decreased proliferation. The factor(s) that mediate cross-talk between TLR3 and Shh signaling cascades are unknown. However, a recent study describing Shh as a direct transcriptional target of NFκB [66] suggests that the NFκB pathway may be pivotal.

Brain structures require Shh for normal proliferation and dorsal-ventral patterning [67]. In humans, loss of Shh signaling causes holoprosencephaly, a birth defect characterized by failure of forebrain development [68], [69]. Conversely, enhanced Shh signaling has been implicated in cancers of prostate, pancreas, muscle and skin as well as the brain [70], [71]. In mice, development of the telencephalon commences at GD11 and peaks between GD14 and 16; this time period is characterized by rapid expansion of NPC followed by generation of neurons through asymmetric divisions [72]. During this critical period of central nervous system development, aberrant TLR3 activation may have profound implications for NPC proliferation, leading to deficits in telencephalon morphogenesis. These findings provide a new mechanistic framework for understanding the pathogenesis of neurodevelopmental disorders associated with gestational and perinatal infections.

## Materials and Methods

### Mice

#### Ethics Statement

All mice were handled in accordance with the Association for Assessment and Accreditation of Laboratory Animals Care international guidelines, with the approval of the Institutional Animal Care and Use Committee at Columbia University under approval ID AC-AAAA4827. The Columbia University IACUC reviewed and approved this study under ID AC-AAAA4827.

Wild-type (WT) pregnant C57BL/6 mice at gestational day (GD) 14 were obtained from Harlan Laboratories (Dublin, VA). Congenic TLR3 deleted mice (TLR3^−/−^) on a C57BL/6 background were obtained by repeated breeding of heterozygous B6;129S1-Tlr^tm1 Flv/J^ TLR3 knockout mice (Jackson Laboratories, Bar Harbor, ME) with WT C57BL/6 for ten generations. Heterozygous mice, carrying the TLR3 deletion were identified at each generation by PCR analysis of total genomic DNA using the following primer sets: NEO_F CTTGGGTGGAGAGGCTATTC, NEO_R AGGTGAGATGACAGGAGATC, TLR3_F ACTCCTTTGGGGGACTTTTG and TLR3_R CAGGTTCGTGCAGAAGACAA. After 10 generations, heterozygous mice were inter-crossed, and congenic homozygous TLR3^−/−^ mice on a C57BL/6 background were selected to maintain the colony. Time-pregnant TLR3^−/−^ mice were obtained by selecting vaginal plug positive (GD 0) dams from breeding pairs of homozygous TLR3^−/−^ mice.

### Primary Neurosphere Cultures

Embryonic cortical neurosphere cultures were obtained using the method of Reynolds and colleagues [8], [9] with minor modifications. Embryos were obtained from cesarean sections of GD14 pregnant dams sacrificed under anesthesia. The embryos were sacrificed by decapitation and the dorsal telencephalon was dissected, trimmed and stripped of meninges in cold Hanks solution containing 5 mM HEPES at pH.8.0, penicillin/streptomycin (Invitrogen, Carlsbad, CA) and 0.8% D-glucose (Sigma-Aldrich, Saint Louis, MO, USA). The tissue was then mechanically dissociated by gentle trituration with fire-polished, glass Pasteur pipettes to yield single cell suspensions. The cells were incubated to generate primary neurospheres in NeuroCult NSC proliferation medium supplemented with 20 ng/ml of human epidermal growth factor (EGF) and Neurocult NSC proliferation supplements (StemCell Technologies, Vancouver, BC, Canada). The cell suspensions were incubated at 37°C, 5% CO_2_ atmosphere in 10 ml at a concentration of 1×10^5^ cells/ml in T-25 tissue culture flasks and left undisturbed for 7 days to generate neurospheres. For experiments with recombinant Shh protein or cyclopamine, cells were cultured in NeuroCult NSC proliferation media containing 5 ng/ml of EGF and supplemented with either 5×nM of recombinant human Shh (Invitrogen, San Diego, CA) or 5 µM of cyclopamine (Toronto Research Chemicals Inc., Ontario, Canada).

### PIC treatment

Polyinosinic-polycytidylic potassium salt (Sigma Aldrich; St. Louis, MO, USA) dissolved in PBS was heated to 55°C for 5×min and allowed to cool at room temperature. dsRNA concentration was measured by UV spectroscopy [65]. Neurosphere cultures maintained in proliferation medium for 7 days were treated with 50 µg/ml of PIC for the last 24 hours of the culture period. We observed robust phenotypic changes in neurosphere cells that were treated with 50 µg/ml of PIC without inducing significant loss of viability; hence, all phenotypic testing was performed with cultures treated with this dose of poly I:C..

### Antibodies

Primary antibodies included: mouse anti-BrdU (1∶50, Clone: B44, Becton Dickinson, San Jose, CA), mouse anti-PAX6 (1∶100, developed by Atsushi Kawakami and obtained from Development Studies Hybridoma Bank, University of Iowa, Iowa city, IA), mouse anti-TBR2-PE (1∶100, Clone: 21 Mgs8, eBioscience, San Diego, CA), chicken anti-mouse serum TLR3 (1∶500, Sigma-Aldrich, St. Louis, MO), rat serum anti-mouse TLR3 (1∶500, eBioscience, San Diego, CA), mouse anti-Nestin-Alexa Fluor 647 (1∶20, Clone: 25/Nestin, BD Pharmingen, San Jose, CA), mouse anti-Tubulin-β-III-NorthernLights 637 (1∶20, Clone: TuJ-1, R&D Systems, Minneapolis, MN), mouse anti-GFAP-Alexa Fluor 488 (1∶50, Clone: 131-15019, Invitrogen, Carlsbad, CA), mouse anti-oligodendrocyte marker O4-PE (10μl, Clone: O4, R&D Systems), goat anti-mouse Patched (1∶20, R&D Systems), Rat anti-Gli1-FITC (1∶100, R&D Systems), rabbit anti-mouse Phospho-MEK1/2 (1∶100, Cell Signaling Technology, Danvers, MA), rabbit anti-mouse Phospho-ERK (1∶100, Cell Signaling Technology), and hamster anti-mouse CD95-FITC (1∶100, Clone: Jo2, BD Pharmingen). Secondary antibodies included donkey anti-mouse-APC, donkey anti-goat-PE, donkey anti-mouse-FITC, donkey anti rat-FITC, (1∶1000, Jackson Immunoresearch, West Grove, MA), goat anti-rat-PE (1∶100, R&D Systems), and goat anti-rabbit-APC (1∶100, R&D Systems). Flow cytometry isotype controls were PE rat IgG2a κ, FITC rat IgG2a κ, APC mouse IgG2a κ, FITC mouse IgG1 κ, and APC mouse IgG1 κ isotype (BD Biosciences, Bedford, MA).

### Flow cytometry

BrdU was added to cortical neurosphere cultures at 10 µM, 12 hours prior to culture processing. Neurospheres were pelleted by centrifugation at 800 xg for 5 minutes. Cell pellets were then suspended in 2 ml of trypLE stable trypsin replacement enzyme (Invitrogen, San Diego, CA) and incubated at 37°C for 15 minutes. The neurosphere suspensions were pelleted again and resuspended in 2 ml of NeuroCult NSC proliferation media supplemented with 250 U/ml of DNAse I (Sigma-Aldrich, Saint Louis, MO, USA). Single cell suspensions were obtained by trituration with fire-polished glass Pasteur pipettes. Intracellular BrdU was measured using the BrdU Flow Kit (BD Biosciences, Bedford, MA). Cells were resuspended in 50 µl of staining buffer (PBS with 1.0% FBS). Cells were fixed, permeabilized, and treated with DNAse (30 µg per tube) to expose incorporated BrdU. Cells were resuspended in 50 µl of BD Perm/fix buffer containing anti-BrdU-FITC antibodies (1∶50) and relevant antibodies specific for detection of intracellular antigens, followed by staining with secondary antibodies. Cells were washed and resuspended in staining buffer and analyzed by multicolor flow cytometry on a LSRII Analyzer (Becton Dickinson, Franklin Lakes, NJ). After gating to exclude dead cells and debris based on forward and side scatter, data were analyzed using FACS DiVa acquisition software (Becton Dickinson, Franklin Lakes, NJ) and FlowJo6.1 (Tree Star, Ashland, OR).

### Apoptosis assays

Cell suspensions of cortical neurospheres were washed twice with PBS, resuspended in 0.1 ml Annexin V binding buffer (BD Biosciences), and incubated with 5 µl of FITC-conjugated Annexin V (BD Biosciences) and 10 µl of propidium iodide (PI) for 15 min at room temperature. Cells were immediately analyzed by flow cytometry on a FACSCalibur (Becton Dickinson). Data were obtained using CellQuest acquisition software (Becton Dickinson) and analyzed using FlowJo6.1 (Tree Star, Ashland, OR, USA). Cells stained with Annexin V-FITC alone and PI alone were used as controls.

### Statistical analysis

StatView version 5.0.1 software (Windows version; SAS Institute, Cary, NC, USA) was used for all statistical analyses. Mann–Whitney U-tests (independent variable: dose group) were used for group comparisons requiring nonparametric analytic approaches. For all tests, statistical significance was assumed where *p*<0.05.

## Supporting Information

Figure S1 **Phase contrast images of primary neurosphere cultures.** EGF-responsive murine cortical cells, isolated from GD 14 WT embryo, were grown for 1 day or for 7 days in proliferation medium supplemented with 20 ng/ml of EGF. (**a**) Small clusters of cells were visible one day after plating. (**b**) Spherical, bright phase and viable neurospheres were identified after 7 days *in vitro*. Magnification, 10X.
(TIF)

Figure S2 **Percentage of nestin-positive cells at different passages.** Cortical cells were obtained from WT embryos at GD14 and cultured in EGF-containing proliferation medium (20 ng/ml) for 7 days to form neurospheres. Cells were passaged once a week by enzymatic and mechanical re-dissociation and re-plated. (**a**) Representative histograms showing the percentages of nestin^+^cells in primary and (**b**) secondary neurospheres (2^nd^ passage) that were in culture for 7 days. Numbers in gates represent the percentages of nestin^+^ cells. Three independent cell culture assays were performed with cells isolated from embryos (n = 12); data from one representative assay is shown.
(TIF)

Figure S3 ***In vitro*** **differentiation of WT neurospheres.** Dissociated cells from WT primary cortical neurospheres were plated onto poly-D-lysine/laminin-coated six-well plates (Sigma) and differentiated in serum-free DMEM/F-12 medium (Invitrogen) supplemented with B27 (Invitrogen), Neurocult NSC supplement (Stem cell) and 1% Fetal calf serum (Invitrogen) for 7 days. (**a–c**) During differentiation, neurosphere-derived cells lost their spherical shape and flattened to form a monolayer (bright field, **a**). Immunofluorescence analysis showed that WT neurospheres were multipotent and expressed genes such as βIII-tubulin (red, **b**) or GFAP (red, **c**), markers characteristic for neurons and astrocytes, respectively. WT neurospheres also expressed nestin (green, **b, c)**. Nuclear staining (blue, **b, c**). (**d–f**) Representative histograms showing the percentages of differentiated WT neurosphere-derived cells expressing tubulin-β-III (**d**), O4 (e), and GFAP (**f**). Numbers in gates represent the percentages of each sub-population. Overlays represent the mean fluorescence intensity expressed as a percentage of the maximum expression: ——, cell lineage marker; ——, isotype control.
(TIF)
