# Supplementary figures and images for: Toll-like Receptor 3 Regulates Neural Stem Cell Proliferation by Modulating the Sonic Hedgehog Pathway
Source: PLoS One. 2011 Oct 25;6(10):e26766. doi: 10.1371/journal.pone.0026766 (PMC3201973; doi:10.1371/journal.pone.0026766)

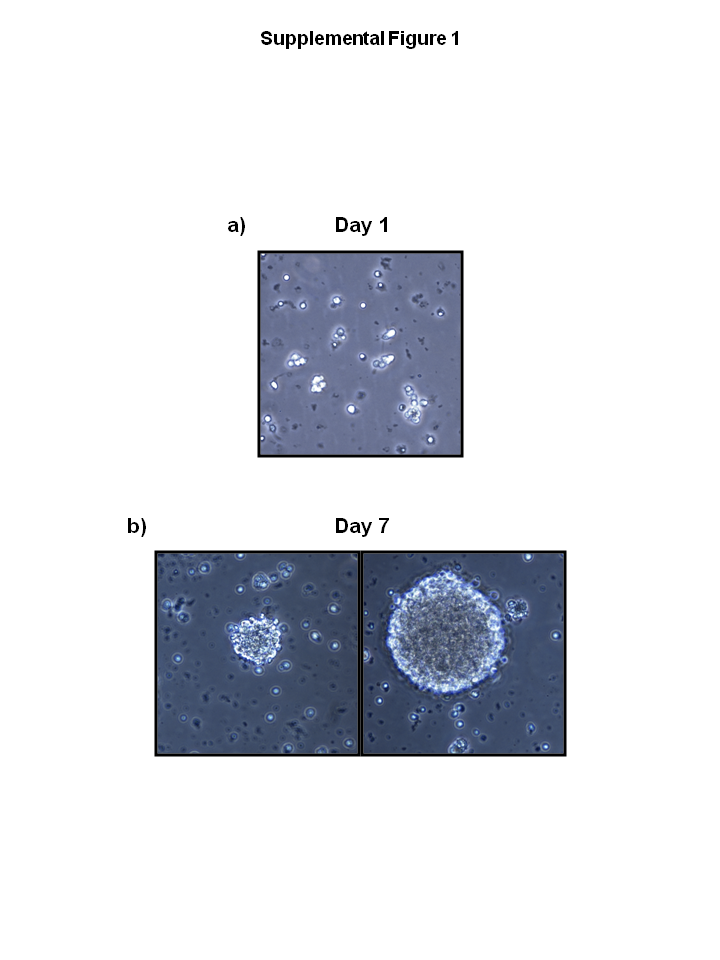

Supplement: Figure S1 — Phase contrast images of primary neurosphere cultures. EGF-responsive murine cortical cells, isolated from GD 14 WT embryo, were grown for 1 day or for 7 days in proliferation medium supplemented with 20 ng/ml of EGF. (a) Small clusters of cells were visible one day after plating. (b) Spherical, bright phase and viable neurospheres were identified after 7 days in vitro. Magnification, 10X. (TIF) [file pone.0026766.s001.tif]

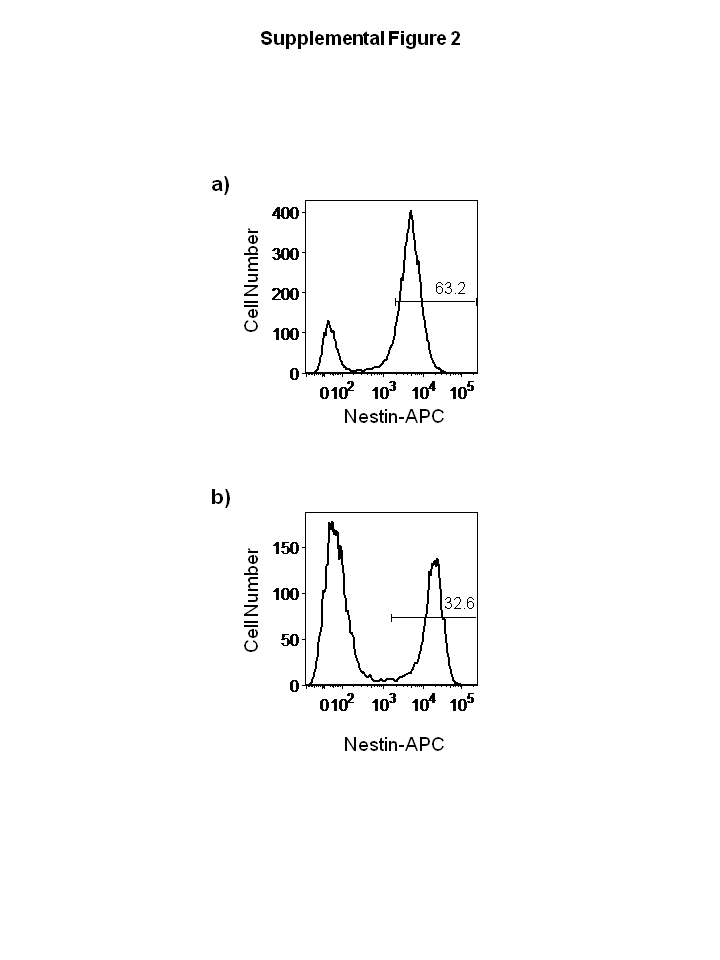

Supplement: Figure S2 — Percentage of nestin-positive cells at different passages. Cortical cells were obtained from WT embryos at GD14 and cultured in EGF-containing proliferation medium (20 ng/ml) for 7 days to form neurospheres. Cells were passaged once a week by enzymatic and mechanical re-dissociation and re-plated. (a) Representative histograms showing the percentages of nestin+cells in primary and (b) secondary neurospheres (2nd passage) that were in culture for 7 days. Numbers in gates represent the percentages of nestin+ cells. Three independent cell culture assays were performed with cells isolated from embryos (n = 12); data from one representative assay is shown. (TIF) [file pone.0026766.s002.tif]

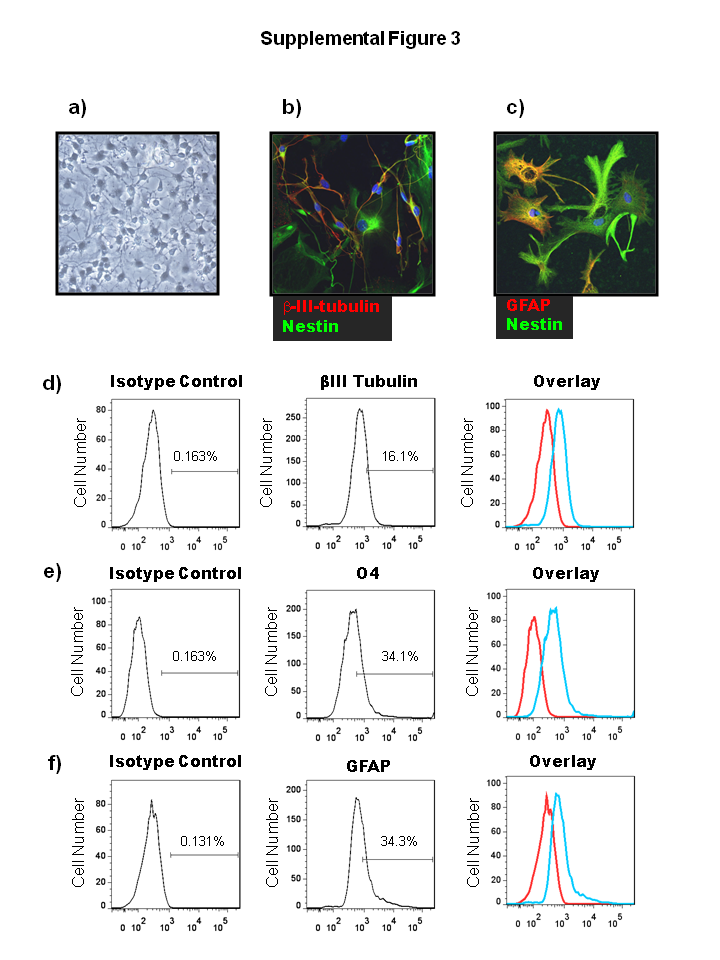

Supplement: Figure S3 — In vitro differentiation of WT neurospheres. Dissociated cells from WT primary cortical neurospheres were plated onto poly-D-lysine/laminin-coated six-well plates (Sigma) and differentiated in serum-free DMEM/F-12 medium (Invitrogen) supplemented with B27 (Invitrogen), Neurocult NSC supplement (Stem cell) and 1% Fetal calf serum (Invitrogen) for 7 days. (a–c) During differentiation, neurosphere-derived cells lost their spherical shape and flattened to form a monolayer (bright field, a). Immunofluorescence analysis showed that WT neurospheres were multipotent and expressed genes such as βIII-tubulin (red, b) or GFAP (red, c), markers characteristic for neurons and astrocytes, respectively. WT neurospheres also expressed nestin (green, b, c). Nuclear staining (blue, b, c). (d–f) Representative histograms showing the percentages of differentiated WT neurosphere-derived cells expressing tubulin-β-III (d), O4 (e), and GFAP (f). Numbers in gates represent the percentages of each sub-population. Overlays represent the mean fluorescence intensity expressed as a percentage of the maximum expression: ——, cell lineage marker; ——, isotype control. (TIF) [file pone.0026766.s003.tif]
